# Particle Accumulation in a Microchannel and Its Reduction by a Standing Surface Acoustic Wave (SSAW)

**DOI:** 10.3390/s17010106

**Published:** 2017-01-07

**Authors:** Yannapol Sriphutkiat, Yufeng Zhou

**Affiliations:** School of Mechanical and Aerospace Engineering, Nanyang Technological University, 50 Nanyang Ave., Singapore Centre for 3D Printing (SC3DP), Singapore 639798, Singapore; yannapol001@e.ntu.edu.sg

**Keywords:** microchannel, clogging, particle accumulation, standing surface acoustic wave (SSAW)

## Abstract

Accumulation of particles in a high concentration on a microchannel wall is a common phenomenon in a colloidal fluid. Gradual accumulation/deposition of particles can eventually obstruct the fluid flow and lead to clogging, which seriously affects the accuracy and reliability of nozzle-based printing and causes damage to the nozzle. Particle accumulation in a 100 μm microchannel was investigated by light microscopy, and its area growth in an exponential format was used to quantify this phenomenon. The effects of the constriction angle and alginate concentration on particle accumulation were also studied. In order to reduce the clogging problem, an acoustic method was proposed and evaluated here. Numerical simulation was first conducted to predict the acoustic radiation force on the particles in the fluid with different viscosities. Interdigital transducers (IDTs) were fabricated on the LiNbO_3_ wafer to produce standing surface acoustic waves (SSAW) in the microchannel. It was found that the actuation of SSAW can reduce the accumulation area in the microchannel by 2 to 3.7-fold. In summary, the particle accumulation becomes significant with the increase of the constriction angle and fluid viscosity. The SSAW can effectively reduce the particle accumulation and postpone clogging.

## 1. Introduction

Inkjet printing has been used widely in recreating a digital image by propelling droplets onto paper, plastic, or other substrates using either continuous or drop-on-demand technology since the late 1970s [1]. Its advantages include low cost and noise, and high resolution. Meanwhile, this versatile computer-aided tool can also be applied in many manufacturing fields with high-throughput, such as the fabrication of functional and structural materials [2], all-polymer transistor circuits [3], organ/tissue printing [4], and recombinant proteins microarrays [5]. However, accumulation and deposition of particles usually occur in nozzle-based printing, especially in small nozzles for extrusion of fine drops. This phenomenon is a progressive process and may cause an obstruction of the upstream fluid flow, either temporarily or permanently, and finally lead to clogging. The clogging problem would result in non-uniformity of the printed part, loss of material, long printing time, and excessive time devoted to printing quality, but it is difficult to predict. For dense micro-particle mixtures, the particle accumulation and the corresponding printability time before the occurrence of clogging are highly dependent on the microchannel geometry and hydrodynamic parameters, such as the fluid viscosity, concentration of micro-particles, and flow rate [6,7]. Therefore, the viscosity and surface tension of bio-ink must be below 40 mPa·s and above 28 mN/m to enable the printing from the tiny nozzle continuously, respectively [8,9]. For reference, the corresponding value of pure water at room temperature and ambient air pressure is approximately 1 mPa·s and 73 mN/m, respectively. However, it is difficult to build a three-dimensional scaffold freeform structure with low viscosity and mechanical strength. Thus, there is a great need to reduce the clogging problem in the nozzle-based printing system and increase its printability.

Obstruction formed by particles can be categorized into three types: complete blocking, arch formation, and deposition clogging [10]. The complete blocking occurs when the particle is larger than the microchannel, common in filtration or sieving. The piling of particles layer by layer would gradually reduce the pressure [11]. When many particles in the stabilized forward flow pass through the microchannel simultaneously, they may get stuck in the microchannel to establish an arch [12], mostly in high particle concentration regions. Clogging is a consequence of gradual deposition and accumulation of particles on the microchannel wall. As a result, the flowing fluid will become gradually narrower or the stream will shrink. Particle accumulation is mostly found near a constriction area due to the retardation effect [13]. The streamlines bend near the constriction, and then the deflection of the particle trajectory would cause the particles to be captured on the wall permanently.

Until now there have been few methods to effectively reduce clogging during the nozzle-based printing. To reduce the interaction force between the liquid and solid layer and the surface tension of printing material, a surfactant is usually added. However, surfactants could change the properties of the cell membrane and decrease cell proliferation. The printed Hep G2 hepatocytes onto hydrogels with the addition of 0.05% pluronic (a biocompatible and Food and Drug Administration approved surfactant) decreased the cell viability from >95% after two days to 50% over 13 days [14]. Electromagnetic force generated by either injecting a DC current or electromagnetic induction (i.e., 1000 A at 5000 Hz) can modify the turbulent flow in the nozzle entry region and reduce the recirculation zone in a cylindrical tundish nozzle and, subsequently, the potential of trapping oxide particles for clogging [15].

Another solution is an acoustic approach, such as using bulk acoustic waves (BAW) and surface acoustic waves (SAW) or travelling SAW (TSAW) which have been applied for microparticle/cell sorting, separation [16,17,18,19,20,21,22], and encapsulation [23,24] in the microfluidic channel. A typical BAW-based microfluidic channel is made of silicon and glass, which are challenging to implement with the fast-protoyping method. Standing waves that are obtained from the leakage of surface acoustic waves into the microchannel from a pair of SAWs propagating in the opposite directions has promising results in cell/particle manipulation with many configurations [25] (i.e., longitudinal particle alignment [26,27], cell separation using tilted-angle standing surface acoustic wave (SSAW) [28], and two- or three-dimensional patterning [29,30,31]). By setting the width of the microchannel to be half of the wavelength, the pressure node of the SSAW can be located along the central axis of the microchannel for the particle accumulation. Thus, it is reasonably hypothesized that the acoustic radiation or acoustophoretic force applied to the particles and the subsequent motion may decrease the deposition of particles on the microchannel wall or even break the bonding between the already-deposited small and isolated particles and the microchannel wall. Standard micro-electro-mechanical systems (MEMS) and soft-lithography procedures permit easy fabrication, miniaturization, and integration of SSAW, making it highly cost-effective for mass production. In addition, characteristics of SSAW-induced particle manipulation can be adjusted by tuning the applied power, wavelength, flow rate, and microchannel geometry.

In this study, the accumulation behavior of particles in a water and hydrogel solution in a poly-dimethylsiloxane (PDMS) microchannel with varied constriction angles was observed under light microscopy in order to understand the mechanism of clogging. The accumulation area was used to quantify the amount of particle deposited. The effect of the acoustic radiation force on the particle in the microchannel with different hydrodynamic parameters (i.e., fluid viscosity and channel geometry) was numerically simulated. Then, a pair of IDTs were fabricated on the piezoelectric substrate (LiNbO_3_) to generate SSAW in the PDMS microchannel. The excitation of SSAW was found to reduce the area of particle accumulation and postpone the onset of clogging. The performance of SSAW was further evaluated at varied alginate concentrations (fluid viscosity) and constriction angles.

## 2. Methodology

### 2.1. Numerical Simulation

For a fluid with a low Reynolds number (i.e., *Re* = 0.55) and Mach number, assuming the fluid to be incompressible, the motion of incompressible laminar flow can be described by [32]:

(1) $$\rho_{0}\left(\vec{u}\cdot \nabla \right)\vec{u}=\nabla \cdot \left[-pI+\mu \left(\nabla \vec{u}+{\left(\nabla \vec{u}\right)}^{T}\right)\right]+\vec{F}\;\rho_{0}\nabla \cdot \left(\vec{u}\right)=0$$

where $\vec{u}$ is the fluid velocity, μ is the dynamic viscosity, $\rho_{0}$ is the fluid density, p is the pressure in the fluid, I is the identity matrix, and $\vec{F}$ is an external force vector. Due to the velocity differences between the fluid and particle, the Stokes drag force produced on the object in the fluid is given by [33]:

(2) $$F_{Drag}=6\pi \mu r\;\left(v_{fluid}-v_{particle}\right)$$

where *r* is the radius of a sphere, and *v_fluid_* and *v_particle_* are the velocities of fluid and particle, respectively.

Acoustophoresis is due to the difference in momentum flux around the particle by the acoustic waves [34]. The wave equation can be described by the total potential velocity (*φ_total_*) as the sum of the incident and scattering waves (*φ_prop_* and *φ_scat_*, respectively).

(3) $$\nabla^{2}\phi_{total}=\frac{1}{c_{0}^{2}}\frac{\partial^{2}}{\partial t^{2}}\phi_{total}$$

When the acoustic wave propagates through the particle, it will cause the particle to oscillate and pulsate. Oscillation creates the dipole scattering while pulsation produces the monopole scattering. The resultant acoustic radiation force applied on the particle is described using the Gauss’s theorem [35]:

(4) $$F^{rad}=\frac{4}{3}\pi r^{3}\nabla \left[f_{mono}\frac{1}{2}k_{0}p_{prop}^{2}-f_{dip}\frac{3}{4}\rho_{0}v_{prop}^{2}\right]\;f_{mono}=1-\frac{k_{p}}{k_{f}},\;f_{dip}=\frac{\rho_{p}-\rho_{f}}{\rho_{p}+\rho_{f/2}}$$

where $\rho_{p}$ and $\rho_{f}$ are the density of particle and fluid, $k_{p}$ and $k_{f}$ are the compressibility of particle and fluid, $f_{mono}$ and $f_{dip}$ are the dimensionless scattering coefficients for monopole and dipole, respectively, and $k_{0}$ is the acoustic wave number.

Numerical simulations were performed on a PC (3.2 GHz, 6 GB memory) using finite element method (FEM) software (COMSOL 5.0, COMSOL, Inc., Burlington, MA, USA). There are two domains in the model, PDMS and water. The fluid flow is assumed to be a fully developed laminar flow with a Reynolds number of 0.55. Drag and acoustophoretic forces were solved in the fluid field in the stationary condition and acoustic field in the frequency domain, respectively. The corresponding motion of particles complies with Newton’s second law. Table 1 lists the material properties used in this simulation.

### 2.2. Experiment Setup

**Figure 1:**
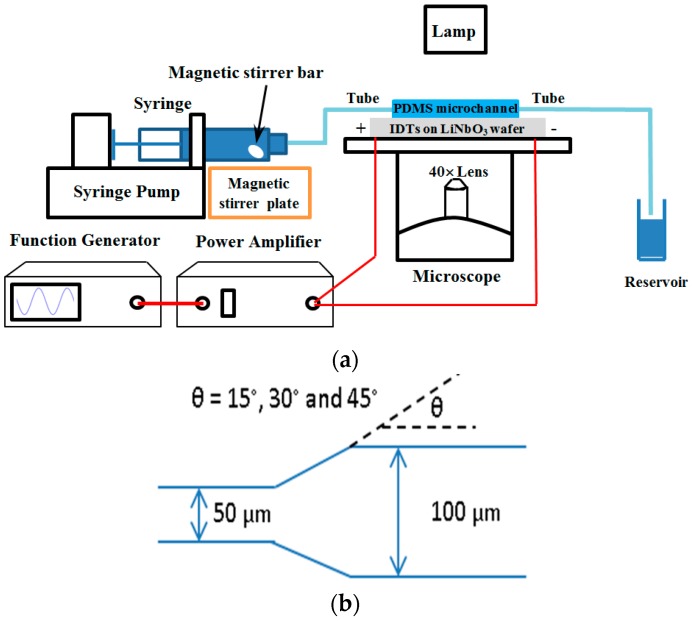
(**a**) Schematic diagram of the experimental setup; and (**b**) the geometry of microchannel with the various constriction angles.

The experimental setup is shown in Figure 1. Microchannels were fabricated using soft-lithography techniques. PDMS (Sylgard 184, Dow Corning, Midland, MI, USA) was mixed with an elastomer base in a ratio of 10:1. The mixture was degassed in a vacuum oven (3608-1CE, Thermo Scientific, Waltham, MA, USA) and poured on a silicon wafer (SI8PSPD, Bonda Technology, Singapore) with a negative tone photoresist (SU-8, Microchem, Westborough, MA, USA) pattern on the top. Then the patterned silicon wafer was degassed again and heated at 70 °C for 3 h in an incubator (BD 56, Binder, Bohemia, NY, USA) for solidification. The length, width, and height of the microchannel is 1 cm, 50–100 μm, and 30 μm, respectively. The polyethylene tubing with an inner diameter of 1 mm was inserted into the microchannels to supply the circulation. Since the cross-sectional area of tubing is significantly larger than that of the microchannel, the hydrodynamic resistance can be neglected. The microparticles (SiO_2_MS-7.75, 8–10 μm, Cospheric, Santa Barbara, CA, USA) were mixed with deionized (DI) water. In order to increase the solution viscosity, sodium alginate powder (180947, Sigma-Aldrich, Singapore) was diluted in DI water by heating to 80 °C and stirred. As sodium alginate is the common hydrogel [36], its concentration of 1%–5% was used and the viscosity is in the range of 2.54–41.7 cPs under atmospheric pressure (around 3–50 times that of the viscosity of water). Before each experiment, the solution was spun by vortex (Barnstead Thermolyne Vortex, Dubuque, IA, USA) for 5 min and then put in an ultrasound sonicator (8892, Cole-Parmer, Vernon Hills, IL, USA) for 15 min to disrupt any agglomeration and achieve a uniform distribution of microparticles. Then the mixture and a small magnetic bar (Z329207, Sigma-Aldrich) was filled into a 3 mL syringe that was driven by a syringe pump (NE-1000, New Era Pump Systems, Farmingdale, NY, USA) at a flow rate of 4 μL/min. The dynamic behavior of microparticles in the microchannel was observed under a light microscope (CKX-41, Olympus, Tokyo, Japan) using 40× magnification and captured by a digital camera (DP70, Olympus), from which the images were quantitatively analyzed using ImageJ software (National Institute of Health, Bethesda, MD, USA). Accumulation area was used to describe the behavior of microparticles up to 30 min or until complete obstruction.

**Figure 2:**
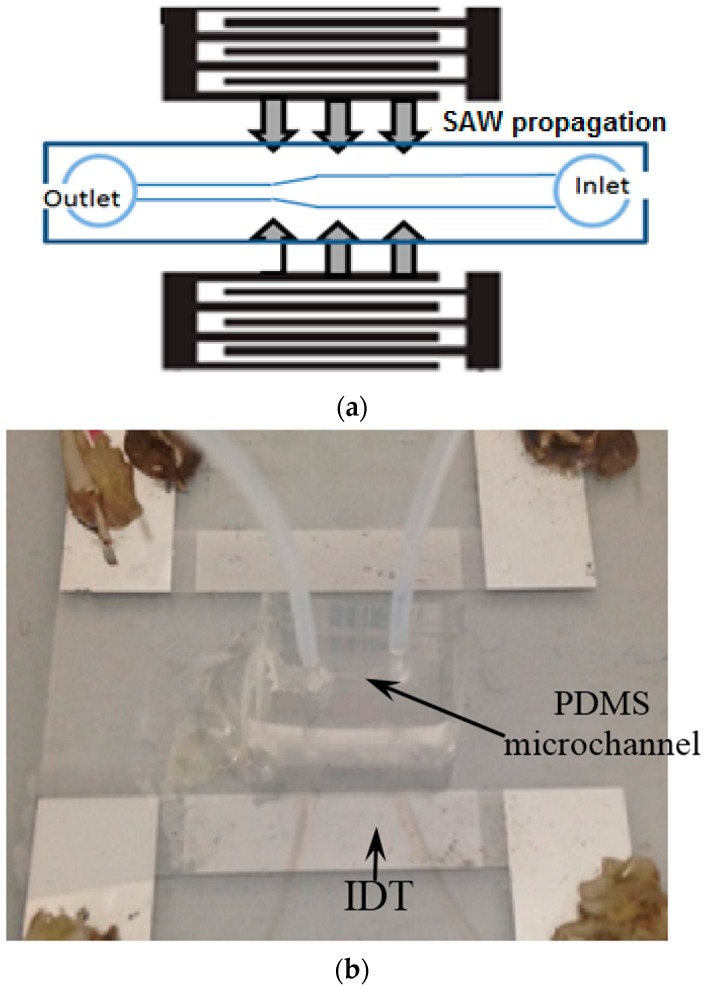
(**a**) Schematic diagram; and (**b**) photo of the SAW propagation through a PDMS microchannel with a constriction area in the experiment.

In order to reduce clogging in the microchannel, a pair of IDTs were fabricated to generate the SSAW (see Figure 2). Twenty nanometers of Cr and 400 nm of Al were deposited on a substrate of a four-inch double-side-polished LiNbO_3_ wafer (Y-128° propagating, University Wafer, Boston, MA, USA). Twenty strips with a width of 50 μm and 2 cm aperture were patterned on the plastic mask (Infinite Graphics, Singapore) for photolithography by coating the positive photoresist (AZ 9260, Microchemicals, Ulm, Germany) on the LiNbO_3_ wafer. Eventually, the Cr-Al layer on the non-exposed area was removed by acetone. Oxygen plasma (Harrick Plasma, Ithaca, NY, USA) was used to treat the surface of PDMS and LiNbO_3_. PDMS was aligned on the LiNbO_3_ and heated at 80 °C in the vacuum chamber. The IDTs were driven by sinusoidal waves at their resonant frequency of 19.95 MHz from a function generator (AFG3000, Tektronix, Beaverton, OR, USA) and then amplified by a power amplifier (0.3–1.0 W, 25A250A, Amplifier Research, Souderton, PA, USA). In order to maximize the power conversion, the impedance of the IDTs was tuned to about 50 Ω using an impedance matching unit built in the lab.

### 2.3. Statistical Analysis

To determine the statistical difference between the testing groups, an analysis of variance (ANOVA) was conducted in SigmaPlot (v11, Systat Software, San Jose, CA, USA). The level of statistical significance was fixed at *p-*value < 0.05 (95% confident interval). At least nine samples were used in each experimental set.

## 3. Results

### 3.1. Numerical Simulation

**Figure 3:**
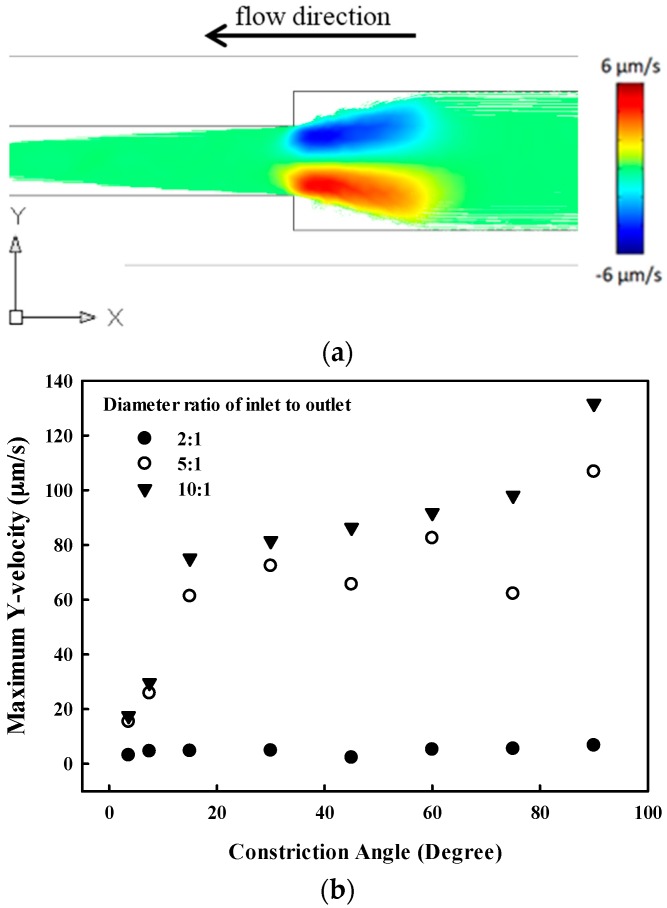
(**a**) Y-velocity of particle under laminar flow in the microchannel at the constriction angle of 90°, inlet of 100 μm, and outlet of 50 μm; and (**b**) maximum velocity under different constriction angles from 3.6° to 90.0° and diameter ratios of the inlet to the outlet at the inlet of 100 μm.

The particle streamline from the inlet to the outlet in the microchannel at the constriction where the streamlines converge is shown in Figure 3. The maximum vertical velocity increases with the angle of constriction. In addition, it is also sensitive to the diameter ratio of the inlet to the outlet, increasing from 6.75 μm/s at 2:1 to 131.7 μm/s at 10:1 at a constriction angle of 90°.

**Figure 4:**
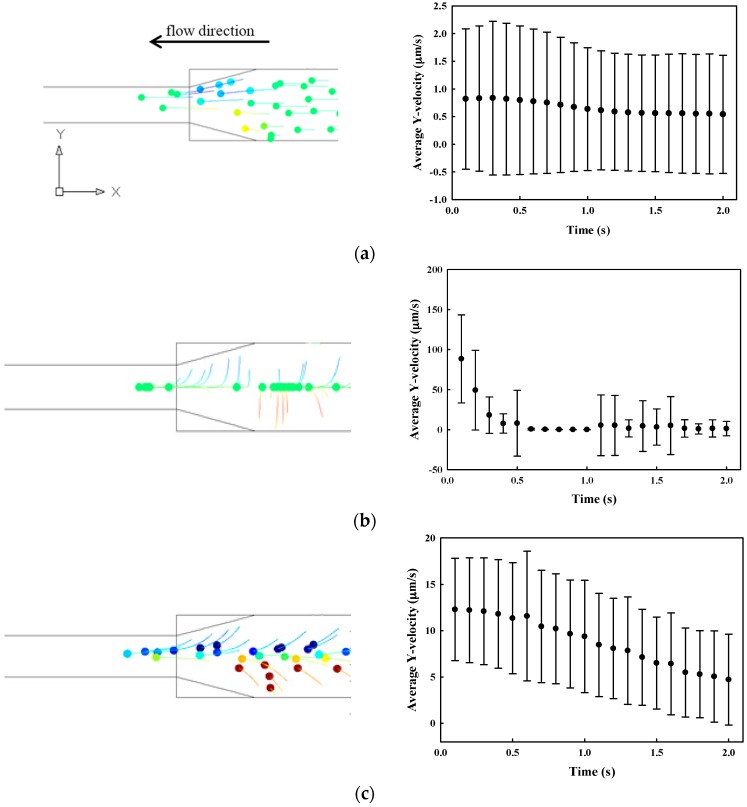

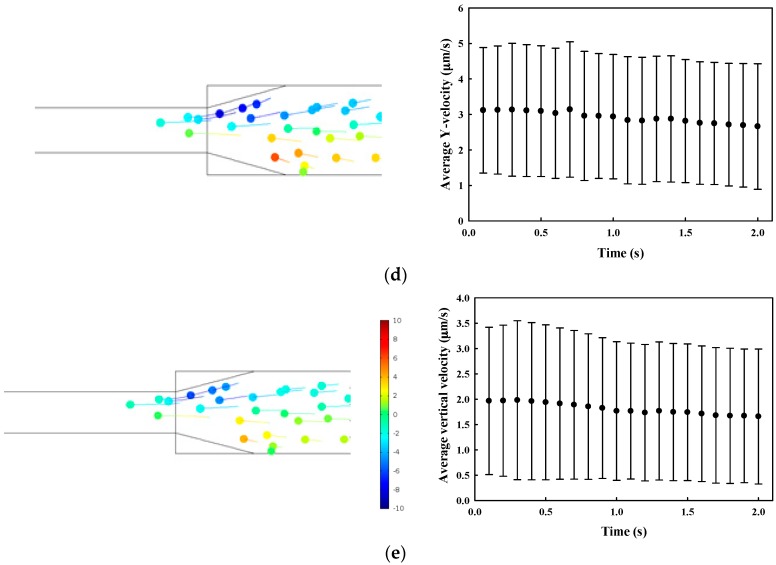
The motion of 30 particles at *t* = 1.2 s (left column) in top view and the Y-velocity of particles presented as mean ± standard deviation (STD) in μm/s (right column) in a 100 μm microchannel at the constriction angle of 15° with the fluid viscosity of (**a**) 8.9 × 10^−4^ Pa·s (1×) without acoustic excitation; and (**b**) 8.9 × 10^−4^ Pa·s (1×); (**c**) 8.9 × 10^−3^ Pa·s (10×); (**d**) 4.45 × 10^−2^ Pa·s (50×); and (**e**) 8.9 × 10^−2^ Pa·s (100×) with the actuation of the standing surface acoustic wave at the vibration amplitude of 0.94 nm.

Particle trajectory in the field of SSAW was simulated by considering both drag and acoustophoretic force. The motion of particles towards the center of the microchannel is dependent on the fluid properties (i.e., dynamic viscosity and density), particle properties (i.e., radius and shape), and particle position. Both microchannel walls are considered as impedance boundaries where planar acoustic waves were reflected. Meanwhile, the inlet and outlet are considered as open boundaries for acoustics. At a vibration amplitude of 0.94 nm, the maximum magnitude of standing wave in the microchannel is 0.18 MPa, and the pressure node is at the center of the microchannel. The motion of 30 microparticles, which are initially distributed uniformly at the inlet, was simulated by a time-transient analysis. The average and standard deviation of Y-velocities of these microparticles at 1.2 s of SSAW activation with the various fluid viscosities from 8.9 × 10^−4^ Pa·s (water viscosity, 1×), 8.9 × 10^−3^ Pa·s (10×), 4.45 × 10^−2^ Pa·s (50×) to 8.9 × 10^−2^ Pa·s (100×), are shown in Figure 4. The particle trajectory in the microfluidic channel are available for view using [37]. The actuation of the SSAW could significantly increase the average Y-velocity of particles in water from 0.8 μm/s to 86.6 μm/s at *t* = 100 ms, which confirms our hypothesis that the acoustophoretic force could effectively push the particles away from the microchannel wall. However, the average Y-velocity, at *t* = 100 ms, decreases with the fluid viscosity of the solution to 12.3 μm/s (10×), 3.1 μm/s (50×), and 1.9 μm/s (100×). The particle motion in a highly-viscous medium (e.g., 100× in Figure 4e), even with the SSAW, is similar to the free motion in water.

**Figure 5:**
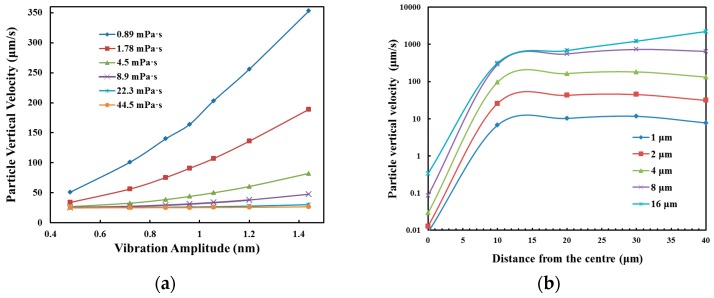
Particle Y-velocity after 10 ms of the SSAW activation under different (**a**) vibration amplitudes and fluid viscosities of 2 μm particles 20 μm away from the microchannel center; and (**b**) distances from the center for different particles at the vibration amplitude of 0.94 nm and a viscosity of 0.89 mPa·s.

The effects of fluid viscosity, the vibration amplitude of the SSAW, particle size, and distance to the microchannel center on the maximum Y-velocity were further investigated (see Figure 5). It is found that the particle Y-velocity increases almost linearly with the vibration amplitude or acoustic pressure, but decreases with the fluid viscosity. In water, the particle Y-velocity is increased by 3.2-fold from 51.1 to 164 μm/s with an increment of vibration amplitude by two-fold (from 0.48 to 0.96 nm). With the increment of fluid viscosity by two-fold (from 0.89 mPa·s to 1.78 mPa·s), the particle velocity is reduced from 164 to 91 μm/s. Therefore, high acoustic power is required to push particles in the highly-viscous medium. In addition, the Y-velocity increases almost linearly with the distance away from the microchannel center and then becomes saturated with small and slow oscillations at about 10 μm. The maximum Y-velocity of the 1-μm particle is 9 × 10^−3^ μm/s and 6.7 μm/s at 0.01 μm and 10 μm away from the center, respectively. Large particle results in fast motion towards the pressure node at the microchannel center, Y-velocity of 10 μm/s and 163 μm/s for 1-μm and 4-μm particle at 20 μm away from the center, respectively.

### 3.2. Experiment Results

Particles (concentration of 1%) flew in the microchannel at the varied constriction angles of 15°, 30°, and 45°, with alginate concentrations of 0% (pure water), 3%, and 5%, and then the corresponding particle accumulation area was measured.

#### 3.2.1. Particle Clogging

**Figure 6:**
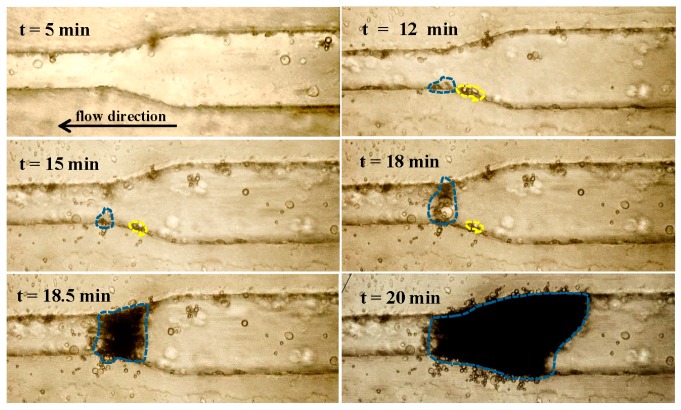
The representative photos of gradual microparticle clogging around the constriction region (15°) in a microchannel with an inlet of 100 μm and outlet of 50 μm. The blue dashes represent the area of permanently-deposited particles while the yellow dots show the detachment of initially-deposited particles at *t* = 18.5 min.

**Figure 7:**
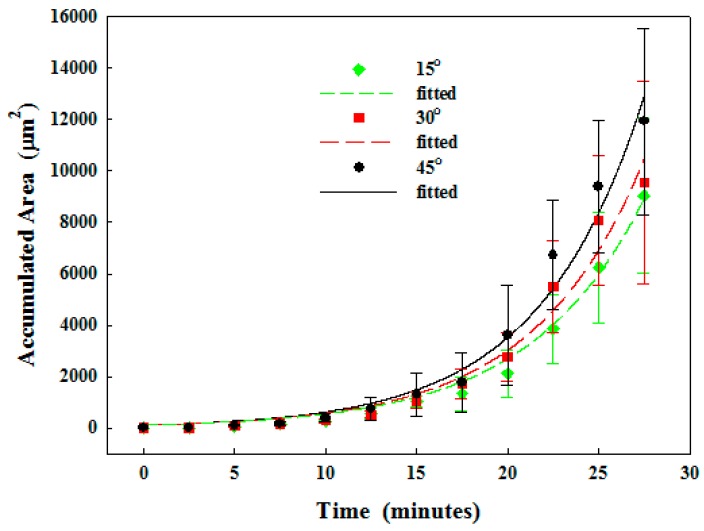
Time-dependent accumulation area of particles in the microchannel at a constriction angle of 15°, 30°, and 45°, and 1% microparticle concentration in deionized water.

Formation of particle clogging in the microchannel was monitored, and the deposition was found to start at about 12 min (see Figure 6). The blue dashed line surrounds the particles deposited at the microchannel constriction. However, some deposited particles were not stable, and so they were detached from the wall (shown as yellow dots at about 18 min). In contrast, these stable particles at the constriction expanded continuously and quickly (at 18–18.5 min). Eventually, the microchannel was almost blocked, particles accumulated rapidly towards the inlet, and the clogged area was densely packed. Overall, the growth of particle accumulation area over time can be fitted exponentially by $Ae^{Bt}$, where *t* is the time (see Figure 7 and Table 2). The accumulation area is initially quite small and then increases significantly after approximately 12 min. It was found that a small constriction angle results in a smaller accumulation area and a delay of clogging. At 25 min of circulation, the accumulation area in the microchannel at the constriction angle of 45° and 15° is 9.4 × 10^3^ ± 2.6 × 10^3^ µm^2^ and 6.3 × 10^3^ ± 2.2 × 10^3^ µm^2^, respectively, as listed in Table 2.

**Figure 8:**
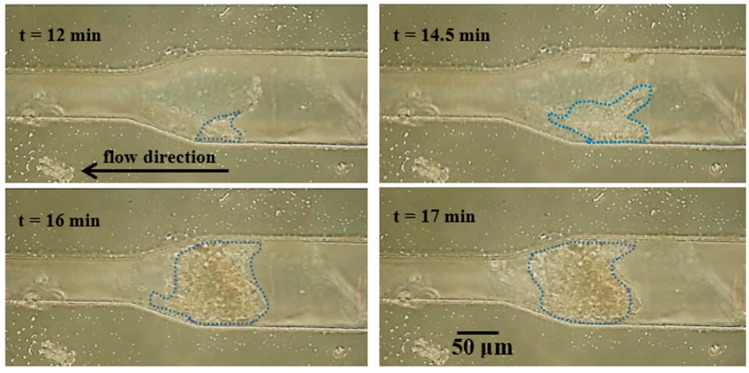
The clogging process of 5% alginate solution in the microchannel at the particle concentration of 1% and the constriction angle of 15°. The blue dotted line contours the agglomerated particles.

**Figure 9:**
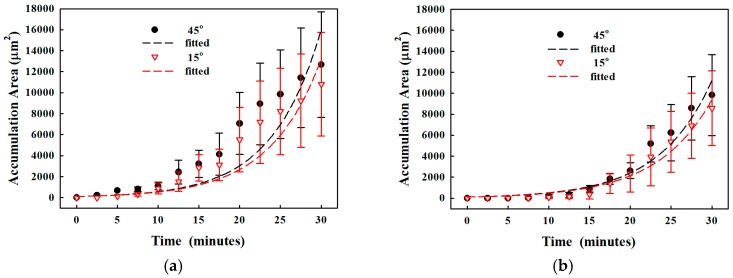
Time-dependent accumulation area of particles in the microchannel at the constriction angle of 15° and 45° with 1% microparticles in (**a**) 3%, and (**b**) 5% alginate solution.

Fluid viscosity affects the particle accumulation. The progressive particle clogging in 5% sodium alginate with 1% particle is shown in Figure 8. It is found that particles and alginate tended to form a lump. Accumulation on the wall occurred before clogging, but the deposited lumps on the wall did not expand until 12 min. The large lump of particles and alginate extended towards the constriction and got stuck at 14.5 min, showing the occurrence of clogging. However, the microchannel has not been fully clogged yet and particles can still flow to the outlet through the opposite wall. After that, the lump continuously grew at the constriction with the deposition of more particles and the increase of its density (darkening in the image) and finally formed the complete clog at 17 min. The progressive growth of accumulation of 3% and 5% of alginate solution with 1% of particles in the microchannel at the constriction angle of 15° and 45° is shown in Figure 9.

#### 3.2.2. Reduction of Clogging by SSAW

**Figure 10:**
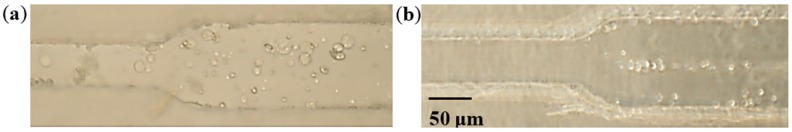
The distribution of microparticles in the microchannel (**a**) before, and (**b**) after, the activation of the SSAW.

**Figure 11:**
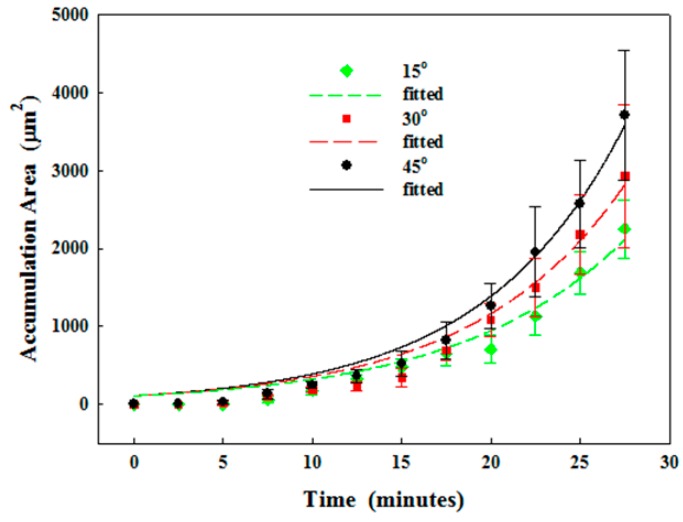
Progressive particle accumulation in the microchannel at the constriction angle of 15°, 30°, and 45° with 1% microparticles in water and actuation of the standing surface acoustic wave (SSAW).

The actuation of the SSAW could push the particles in the microchannel towards the pressure node by the acoustophoretic force, and the location of pressure node is close to the simulation using the excitation frequency of IDTs (see Figure 10). The effect of SSAW on the accumulation area was studied (see Figure 11). The reduction in the accumulation area using SSAW at the varied constriction angles is similar, 3.6 to 3.7-fold (from 9.4 × 10^3^ ± 2.6 × 10^3^ µm^2^ to 2.6 × 10^3^ ± 5.7 × 10^2^ µm^2^ at 45°, from 8.1 × 10^3^ ± 2.5 × 10^3^ µm^2^ to 2.2 × 10^3^ ± 5.0 × 10^2^ µm^2^ at 30°, and from 6.3 × 10^3^ ± 2.2 × 10^3^ µm^2^ to 1.7 × 10^3^ ± 2.7 × 10^2^ µm^2^ at 15°, respectively, *p* < 0.05). Overall, the SSAW was able to delay clogging.

**Figure 12:**
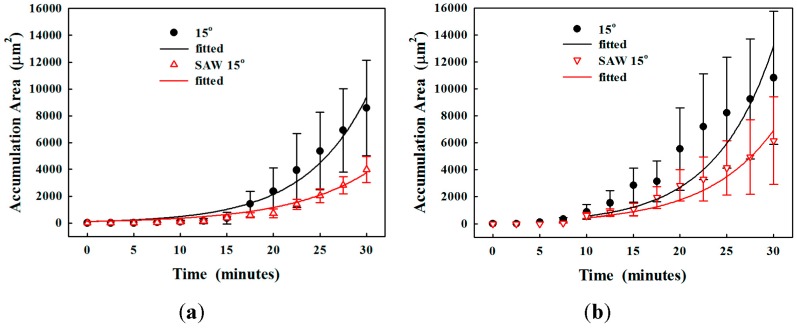
Progressive particle accumulation in the microchannel at the constriction angle of 15° with 1% microparticles in (**a**) 3%; and (**b**) 5% alginate solution, without and with the actuation of the standing surface acoustic wave (SSAW).

SSAW actuation could also reduce the accumulation area for a solution with high viscosity, as shown in Figure 12. Here the constriction angle of 15° was only investigated because the other configurations have a very short time of developing the complete obstruction. The accumulation area of 3% and 5% alginate after 25 min of circulation is 2.0 × 10^3^ ± 5.2 × 10^2^ µm^2^ and 4.1 × 10^3^ ± 2.0 × 10^3^ µm^2^, which corresponds to 2.62- and 1.99-fold reductions, respectively. Statistical analysis showed a significant reduction in the accumulation area by the actuation of the SSAW (*p* = 0.003 and 0.019, respectively).

## 4. Discussion and Conclusions

The behavior and deposition of microparticles in a microchannel and the formation of clogging were observed under the light microscope. It is found that the particle deposition begins at isolated locations on the channel wall, followed by the accumulation of more particles and the coalescence of multiple accumulation sites. Once the growing accumulation from both sides of the microchannel wall make contact with each other, the flow blocking (maybe partial obstruction) will occur. Afterward, the accumulation extends toward the inlet, and its density increases for the complete obstruction. The progressive growth of the accumulation area can be fitted by an exponential curve (*R*^2^ > 0.9), and increases with concentration of alginate and the constriction angle. In order to reduce the particle accumulation and postpone clogging, SSAW in the microchannel was proposed and evaluated. Significant reduction in the accumulation area was found (2.0 to 3.7-fold) regardless of the constriction angle, but decreases with the concentration of alginate or the fluid viscosity.

The accumulation area of particles and alginate increases over time, but there are several stages during the process. At the initial stage, particles occasionally and randomly deposit on the microchannel wall due to the attractive force from the solid boundary. Particles with high zeta potential are stabilized while those with low value tend to coagulate or flocculate [38]. The accumulation area is small and grows very slowly. Then the attractive force becomes larger with more deposited particles, and the accumulation area increases almost linearly. After that, the accumulation area increases exponentially, which may be due to several reasons. One is fluid blockage near the constriction. Initial expansion of deposited particles is permeable and allows the liquid to pass through, but traps the particles. Then the structure is packed so densely that the microchannel is completely clogged [39]. Another reason is that the van der Waals force and the deflection of the streamline from the deposited particles and the microchannel wall could overcome the electrostatic barrier to capture more incoming particles [10,40,41]. When the particles slide over the deposition layer, the induced shear field reduces their velocities along the wall so that they will aggregate with the deposited particles. Thus, initially deposited particles may work as an accelerator in the particle accumulation [10,42]. It is reasonably hypothesized that the reduction in the initial deposition would postpone the accumulation effectively, but may not completely avoid it. However, the large aggregate is not always stable due to the particle detachment. When the rolling moment derived from the fluid overcomes the rolling resistance, the hydrodynamic detachment of colloids will occur [43]. Once the aggregate expands and connects with the others or the opposite microchannel wall, clogging will occur. Then particles accumulate dramatically towards the inlet, and the density of accumulation increases correspondingly owing to the compressed inter-particle space.

**Figure 13:**
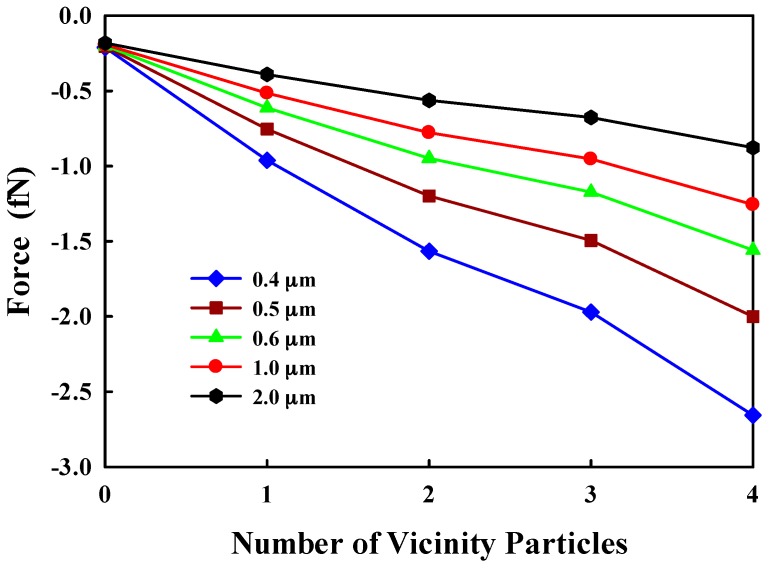
The attractive force on an 8-μm suspending microparticle from the wall and deposited particles at the various distances.

The inter-particle force is critical for the particle accumulation and could be estimated by the Derjaguin–Landau–Verwey–Overbeek (DLVO) theory. The attractive force between the wall and suspending 8-μm particle, and between the deposited particles and suspending ones, is shown in Figure 13, assuming that the deposited particles are located beside each other on the same layer and the suspending particle can contact with only a few deposited particles [10], which are valid at low ionic strength [6,39]. The attractive force increases with the number of deposited particles and the closeness between them. Thus, the initial particle deposition could generate the attractive force for the accumulation of more particles, and an effective approach to reducing the accumulation should be performed at the initial stage of this phenomenon.

Particle deposition was usually found near the constriction of the microchannel [10,42,44]. A small constriction angle achieves less particle accumulation, which may be due to the small forward flow stagnation surfaces available and the high permeability by inevitable funneling of particles into the constriction [39]. Low Y-velocity facilitates the streamline to converge almost linearly with less fluctuation, which may reduce the trapping probability of the particles. The supporting force from the microchannel wall at the constriction on the deposited particles should be greater than the drag force, which increases with the constriction angle, to avoid the detachment. The surface property also plays an important role in this phenomenon. The interaction energy barrier is significantly small for rough surfaces over a large range of relevant particle-wall separation distances to facilitate primary minimum deposition. Similarly, the rough surface has the decreased depth of the primary energy minimum so that the adhered particles are weak and even detach due to hydrodynamic forces and diffusion [45]. In addition, ionic strength of the medium determines the surface interaction between the wall and particle and, subsequently, the formation of clogging. Clogging is slow but robust and dense under low ionic strength while fast, but fragile and loose under high ionic strength [39]. More work is required to fully understand the phenomenon and mechanisms of clogging.

At the high alginate concentration, a large lump of alginate particles is formed because the alginate has a strong intermolecular force for adhesion [46]. The characteristics of clogging in the alginate solution are similar to those in water. Particles flow as single or small aggregates, whereas particles and alginate may form a large lump and deposit on the wall with much higher stability. A 5% alginate solution tends to form aggregates as large as 30 μm. Although alginate solution is highly viscous, it is a shear thinning material, its viscosity decreasing with the applied stress [47]. As extrusion increases the flow rate and normal stress at the tip of a narrow nozzle, the alginate solution has no problem through the nozzle. Thus, this characteristic makes the use of alginate popular in 3D extrusion-based bio-printing. In addition, the biological substance also has a strong intermolecular force for easy aggregation, such as cell adhesion molecules of selectins, integrins, syndecans, and cadherins [48]. Intermolecular interactions have already been utilized to induce the controlled assembly of macroscopic objects, such as molecular targeting using covalent bonding (dissociation energy of 30–260 kcal/mol), drug incorporation of the therapeutic agent with hydrogel, cell spheroids for pharmaceutical screening, and the investigation of cancer metastasis [49]. This suggests that the clogging problem in 3D bio-printing may be more serious than that of microparticles investigated here.

SSAW has shown a significant reduction in particle accumulation in water and low alginate concentration medium because of two reasons. First, particles are pushed towards the pressure node (i.e., center of the microchannel) by acoustophoresis, whose force should be much larger than the van der Waals force from the wall. If both electrostatic and Born’s repulsion forces are included, the pushing force will be even larger, but these interparticle forces are weak at a large distance [50,51]. Second, the acoustic streaming generates viscous torque for the rotation of particles along the interphase boundaries [52,53]. Such rotation may be able to reduce the aggregation between flowing particles and deposited ones on the wall by the slippery effect [39,54]. The acoustic streaming generated by SSAWs has been used to remove nonspecifically bound proteins [55]. In highly viscous fluid, an additional term should be added to the dipole scattering coefficient as the viscosity-dependent correction by considering Prandtl-Schlichting boundary layer theory and acoustic boundary layer [56]:

(5) $$f_{dip}=\frac{2\left(1-\gamma \right)\left(\rho_{p}-\rho_{0}\right)}{2\rho_{p}+\rho_{0}\left(1+\frac{9}{2}\left[1+i\left(1+\frac{\delta }{r}\right)\right]\left(\frac{\delta }{r}\right)\right)}$$

where δ is the distance to the boundary layer, i is the complex unit. The smaller *r* relative to *δ*, the larger the effect of viscosity. There is a strong enhancement proportional to (*k_0_r*)^−3^ in comparison to the inviscid case due to non-vanishing interference between the incident and scattered waves. However, for SSAW and large nearly neutral buoyancy particles (i.e., cells), the acoustophoretic force in the inviscid medium is negligible (<1%). Therefore, less effect of SSAW in this study is mainly due to the viscous flow.

The motion of microparticles is determined by the resultant action of acoustic radiation and streaming force [57]. SSAW has a higher magnitude of acoustic streaming than the BAW at the same driving frequency, and small particles are dominated by acoustic streaming. Critical particle size is about 10 μm at the actuation frequency of 6.65 MHz where radiation dominates the motion of microparticles [57]. At the frequency of 19.95 MHz as used in this study, acoustic radiation force would become more significant. Acoustic streaming induced by SSAW is found to be relatively strong near the channel side walls due to the inherent travelling wave component and increases with the height of the microchannel [57,58]. Particle deposition mostly occurs where the fluid streamline deflects, such as at the constriction and entrance of the microchannel, along the side wall of the PDMS microchannel. However, only a few particles deposit on the top and bottom surfaces, theoretically. The acoustic streaming may be also beneficial in preventing particle deposition when it circulates particles above the bottom.

In summary, the exponential growth of the accumulation area of particles in a microchannel is determined by both the geometry of the microchannel and the hydrodynamic parameters. The small constriction angle can moderately (~30%) reduce the accumulation area and delay the catastrophic clogging. The concentration of alginate (5%) leads to the lower increase of the accumulation area, but the stagnation of large aggregates at the constriction. A numerical model was established to simulate the particles’ motion by SSAW with the consideration of fluid and particle properties, acoustic attenuation, acoustic impedance, laminar flow, drag, and acoustophoretic forces. The actuation of SSAW can reduce the accumulation area significantly in water by almost 3.7-fold. However, the increase of fluid viscosity (5% of sodium alginate) reduces the improvement of SSAW to two-fold. This acoustic approach provides a low-cost and effective solution to the particle accumulation and could delay clogging in the nozzle-based printing. The transparent microchannel allows the observation of the clogging phenomenon and understanding of the mechanisms. However, extrapolation to the nozzle in practice needs further investigation. Although the acoustophoretic force shows the ability to reduce the clogging here, low acoustic power is usually produced by the current IDTs on rigid substrate for the lesser effect on the highly viscous fluid. However, the use of flexible substrate or thin film with IDTs patterned on can be attached to the nozzle surface and activated at the input power up to 35 W [59,60,61]. The other option is the use of BAW from the curved transducer attached to the nozzle. Interference of the travelling wave and the reflected wave from the other side of the nozzle wall will form the standing wave. Piezoelectric ceramics can withstand high electric power.

## Figures and Tables

**Table 1:** Material properties used in the simulation at the temperature of 27 °C.

Medium	Parameters	Values
Water	density, *ρ_w_*	997 kg/m
	speed of sound, *c_w_*	1497 m/s
	viscosity, *μ_w_*	0.890 mPa·s
	compressibility, *κ_w_*	448 TPa^−1^
Microparticle	density, *ρ_p_*	1050 kg/m
	speed of sound, *c_p_*	2350 m/s
	Poisson’s ratio, *ε_p_*	0.35
	compressibility, *κ_p_*	249 TPa^−1^
Poly-dimethylsiloxane (PDMS, 10:1)	density, *ρ_PDMS_*	920 kg/m
	speed of sound, *c_PDMS_*	1076.5 m/s
Lithium niobate (LiNbO_3_)	speed of sound, *c_LNB_*	3990 m/s
	wavelength, *λ*	200 μm
	frequency, *f*	19.95 MHz

**Table 2:** Time-dependent accumulation area in the microchannel with 1% microparticle concentration in alginate solution fitted by $Ae^{Bt}$ and the accumulation area at 25 min of circulation.

Angle	SSAW	Alginate	Accumulation Area (μm^2^)	A	B	*R*^2^
15°	No	0%	6.3 × 10^3^ ± 2.2 × 10^3^	6.2 × 10^1^	1.8 × 10^−1^	1.00
30°	No	0%	8.1 × 10^3^ ± 2.5 × 10^3^	1.3 × 10^2^	1.6 × 10^−1^	0.97
45°	No	0%	9.4 × 10^3^ ± 2.6 × 10^3^	1.5 × 10^2^	1.6 × 10^−1^	0.98
15°	Yes	0%	1.7 × 10^3^ ± 2.7 × 10^2^	5.0 × 10^1^	1.4 × 10^−1^	0.99
30°	Yes	0%	2.2 × 10^3^ ± 5.0 × 10^2^	5.0 × 10^1^	1.5 × 10^−1^	0.99
45°	Yes	0%	2.6 × 10^3^ ± 5.7 × 10^2^	6.1 × 10^1^	1.5 × 10^−1^	1.00
15°	No	3%	5.4 × 10^3^ ± 2.9 × 10^3^	1.5 × 10^2^	1.4 × 10^−1^	0.97
15°	No	5%	8.2 × 10^3^ ± 4.1 × 10^3^	6.3 × 10^2^	9.8 × 10^−2^	0.95
15°	Yes	3%	2.0 × 10^3^ ± 5.2 × 10^2^	3.9 × 10^1^	1.6 × 10^−1^	0.99
15°	Yes	5%	4.1 × 10^3^ ± 2.0 × 10^3^	2.7 × 10^2^	1.1 × 10^−1^	0.97
45°	No	3%	6.2 × 10^3^ ± 2.7 × 10^3^	2.1 × 10^2^	1.3 × 10^−1^	0.96
45°	No	5%	9.9 × 10^3^ ± 4.2 × 10^3^	9.0 × 10^2^	9.2 × 10^−2^	0.95
